# Detection of Pathogen Exposure in African Buffalo Using Non-Specific Markers of Inflammation

**DOI:** 10.3389/fimmu.2017.01944

**Published:** 2018-01-11

**Authors:** Caroline K. Glidden, Brianna Beechler, Peter Erik Buss, Bryan Charleston, Lin-Mari de Klerk-Lorist, Francois Frederick Maree, Timothy Muller, Eva Pérez-Martin, Katherine Anne Scott, Ockert Louis van Schalkwyk, Anna Jolles

**Affiliations:** ^1^Department of Integrative Biology, Oregon State University, Corvallis, OR, United States; ^2^College of Veterinary Medicine, Oregon State University, Corvallis, OR, United States; ^3^SANPARKS, Veterinary Wildlife Services, Skukuza, South Africa; ^4^The Pirbright Institute, Woking, United Kingdom; ^5^Office of the State Veterinarian, Department of Agriculture, Forestry and Fisheries, Skukuza, South Africa; ^6^Vaccine and Diagnostic Development Programme, Transboundary Animal Diseases, Onderstepoort Veterinary Institute, Agricultural Research Council, Onderstepoort, South Africa; ^7^Department of Microbiology and Plant Pathology, Faculty of Agricultural and Natural Sciences, University of Pretoria, Pretoria, South Africa

**Keywords:** emerging infectious disease, disease surveillance, wildlife, inflammation, haptoglobin, serum amyloid A, IFNγ, TNF-α

## Abstract

Detecting exposure to new or emerging pathogens is a critical challenge to protecting human, domestic animal, and wildlife health. Yet, current techniques to detect infections typically target known pathogens of humans or economically important animals. In the face of the current surge in infectious disease emergence, non-specific disease surveillance tools are urgently needed. Tracking common host immune responses indicative of recent infection may have potential as a non-specific diagnostic approach for disease surveillance. The challenge to immunologists is to identify the most promising markers, which ideally should be highly conserved across pathogens and host species, become upregulated rapidly and consistently in response to pathogen invasion, and remain elevated beyond clearance of infection. This study combined an infection experiment and a longitudinal observational study to evaluate the utility of non-specific markers of inflammation [NSMI; two acute phase proteins (haptoglobin and serum amyloid A), two pro-inflammatory cytokines (IFNγ and TNF-α)] as indicators of pathogen exposure in a wild mammalian species, African buffalo (*Syncerus caffer*). Specifically, in the experimental study, we asked (1) How quickly do buffalo mount NSMI responses upon challenge with an endemic pathogen, foot-and-mouth disease virus; (2) for how long do NSMI remain elevated after viral clearance and; (3) how pronounced is the difference between peak NSMI concentration and baseline NSMI concentration? In the longitudinal study, we asked (4) Are elevated NSMI associated with recent exposure to a suite of bacterial and viral respiratory pathogens in a wild population? Among the four NSMI that we tested, haptoglobin showed the strongest potential as a surveillance marker in African buffalo: concentrations quickly and consistently reached high levels in response to experimental infection, remaining elevated for almost a month. Moreover, elevated haptoglobin was indicative of recent exposure to two respiratory pathogens assessed in the longitudinal study. We hope this work motivates studies investigating suites of NSMI as indicators for pathogen exposure in a broader range of both pathogen and host species, potentially transforming how we track disease burden in natural populations.

## Introduction

Emerging infectious diseases cause human suffering (1, 2), threaten food security (3), and contribute to the decline of vulnerable populations and species (4). As such, in the face of elevated rates of infectious disease emergence in humans (5, 6), domestic animals (7) and wildlife (8–10), effective surveillance for pathogen exposure is increasingly important.

Surveillance for emerging infections is challenging because it requires detection of previously unreported infectious agents, and/or diagnosis of exposure or infection in understudied animal species. Indeed, animals are hosts to hundreds of pathogens and parasites (11) with previously unidentified species regularly documented (12–14). Yet, available disease diagnostics typically target known infections that cause detectable pathology in humans or economically important domestic animals resulting in a relatively narrow range of tests that are highly pathogen specific. Common molecular techniques to detect pathogens include tests that detect genetic material of the pathogen itself and antibody-based diagnostics that detect the host’s antibodies to a given pathogen. Advancing sequencing methods show promise for simultaneously detecting a wider range of pathogens (15, 16) but, while genetically based techniques often have high sensitivity and specificity, they are limited to detection of active infections. Many infections last only a few days and thus may escape detection unless sampling can occur on a tight time frame. Most importantly, diagnostic techniques based on amplifying pathogen genetic material still require pathogen specific primers and/or previous publication of genetic sequences and are, thus, unsuitable in situations where the identity of pathogens is uncertain. Antibody-based techniques, such as enzyme-linked immunosorbent assays or immnuofluorescence assays, offer a way to detect infection after pathogen exposure has occurred because antibody titers to many infections can remain elevated for months to years after primary infection (17). However, antibody-based techniques typically used in disease diagnostics are highly pathogen specific, which limits their utility in detecting novel infections.

An ideal diagnostic approach for monitoring (often unknown) infections in natural populations would complement existing genetic and antibody techniques by detecting the presence of pathogens non-specifically, using immunological markers that indicate recent presence of infection. Ideal markers should increase rapidly and reliably in response to a broad range of pathogens and remain elevated for a consistent period after active infection has subsided. A test that detects exposure both early in infection as well as past pathogen clearance could aid in monitoring population health and improve surveillance for emerging infections.

Here, we suggest that non-specific markers of inflammation (NSMI hereafter) have potential for use in detecting pathogen exposure in natural populations. NSMI include APP [this study: haptoglobin, serum amyloid A (SAA)] and cytokines (here: TNF-α, IFNγ). APP are an integral part of the acute inflammatory response to pathogen exposure and engage in opsonization of pathogens and scavenging of toxic substances (18). SAA is produced by the liver after acute phase induction by pro-inflammatory cytokines; its main functions include binding cholesterol from inflammation sites, modulating the function of innate immune cells, and opsonizing pathogens for destruction by immune cells (18). Haptoglobin binds hemoglobin, which prevents oxidative damage and deprives bacteria of iron needed to grow (18). Cytokines are small “messenger” proteins secreted by immune cells to mediate the immune response. TNF-α is a primary signaling molecule in systemic inflammatory reactions and is a vital component of the acute phase response; IFNγ is a key signaling molecule in clearance of intracellular pathogen infections (19).

**Figure 1:**
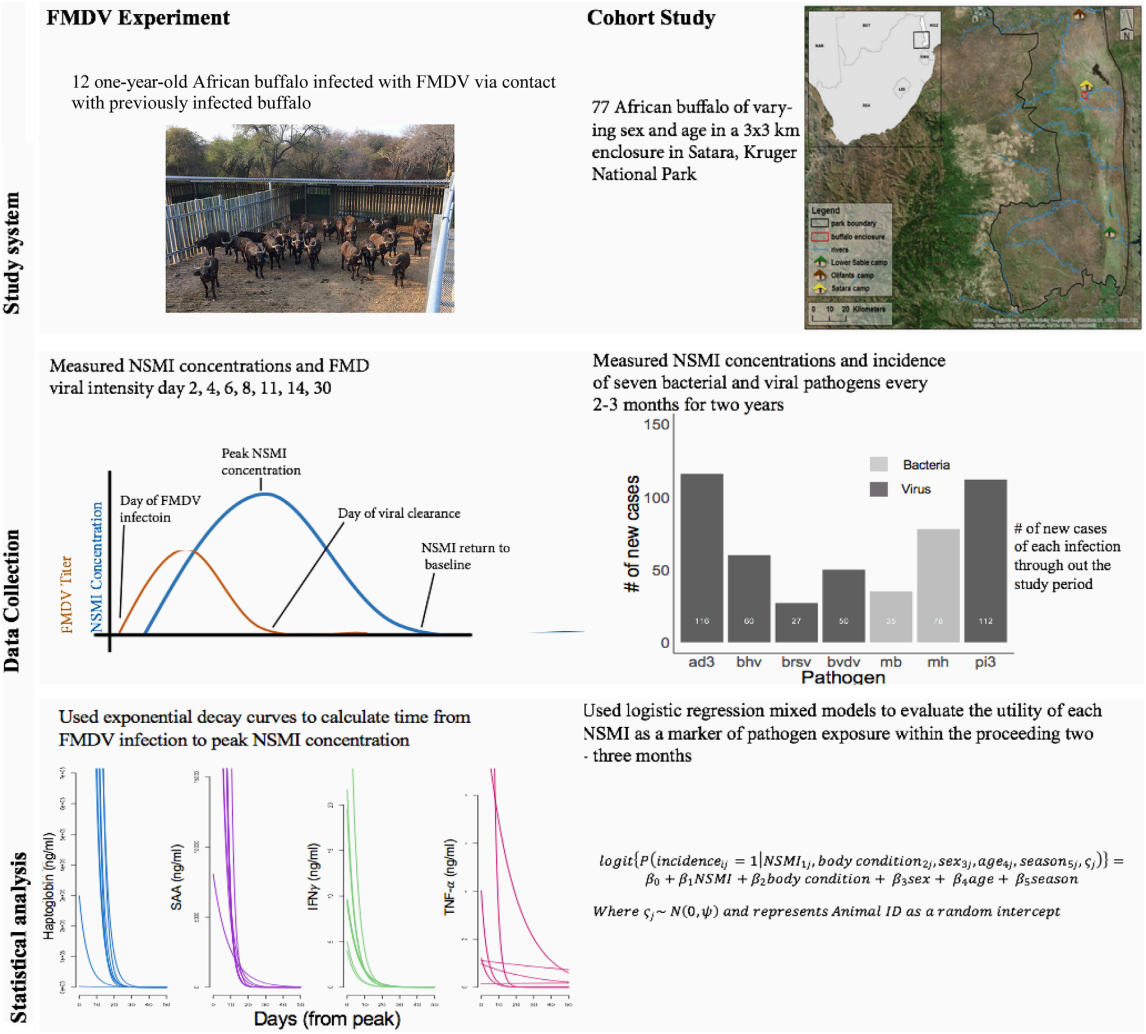
A schematic illustrating the study design and analysis for the foot-and-mouth disease virus (FMDV) experiment and cohort buffalo longitudinal study. The bar graph (cohort study, middle panel) depicts the number of new cases throughout the study period of seven respiratory parasites. The number of new cases of viral parasites are displayed in dark gray (ad3 = Adenovirus; bhv = bovine herpes virus II; brsv = bovine respiratory syncytial virus; bvdv = bovine viral diarrheal virus; pi3 = Parainfluenza virus), the number of new cases of bacterial parasites are displayed in light gray (mb = *Mycoplasma bovis*, mh = *Mannheimia haemolytica*). The line graphs (FMDV experiment, bottom panel) illustrate the exponential decay curve fit from day of peak NSMI concentration to day NSMI returned to baseline for each animal. All animals mounted haptoglobin, SAA, and IFNγ responses, however, only three animals mounted a TNF-α response.

We combined an infection experiment and a longitudinal observational study to evaluate the utility of these four NSMI as indicators of pathogen exposure in a wild mammalian species, African buffalo (*Syncerus caffer*) (Figure 1). Specifically, in the experimental study we asked (1) How quickly do buffalo mount NSMI responses upon challenge with an endemic pathogen, foot-and-mouth disease virus (FMDV); (2) for how long do NSMI remain elevated after viral clearance; and (3) how pronounced is the difference between peak NSMI concentration and baseline NSMI concentration? In the longitudinal study, we asked (4) Are elevated NSMI associated with recent exposure to seven bacterial and viral respiratory pathogens, in a natural host population?

## Materials and Methods

African buffalo (*Syncerus caffer*) included for this study were located within Kruger National Park (KNP), a 19,000 km^2^ reserve located in northeastern South Africa. Two populations were used for the study: (1) 12 1- to 2-year-old bovine tuberculosis (BTB) and FMDV free wild-caught buffalo obtained from Hluwluwe iMfolozi Park and transferred to the Skukuza State Veterinary enclosure (FMDV experiment buffalo, hereafter); (2) a herd of 60–75 wild buffalos, of mixed age and sex, contained within a 900-ha enclosure near Satara camp in the central area of KNP (cohort buffalo, hereafter). The first population was used in a FMDV challenge experiment identifying triggers of FMDV transmission and tracing viral evolution; the second population is part of an ongoing observational study identifying drivers of FDMV dynamics. The study was conducted under South Africa Department of Agriculture, Forestry and Fisheries Section 20 permits Ref 12/11/1 and Ref 12/11/1/8/3, ACUP project number 4478 and 4861, Onderstepoort Veterinary Research Animal Ethics Committee project number 100261-Y5, and the Kruger National Park Animal Care and Use Committee project number JOLAE1157-12 and JOLAE1157-13.

### Field Sampling

#### FMDV Experiment Buffalo

Foot-and-mouth disease virus is an endemic viral infection of cloven-hoofed ungulates, with African buffalo acting as the maintenance host (20). Briefly, 12 buffalo were exposed to FMDV (day 2) by allowing them to mix with recently infected [*via* injection, using protocols optimized previously for buffalo: Maree et al. (21)] animals. All 12 recipient animals were sedated on days 2, 4, 6, 8, 11, 14, and 30 days post FMDV exposure to allow for collection of blood samples for quantification of NSMI and FMD viremia. Immobilizations were conducted by South African State Veterinarians using standard protocols for buffalo (22). Blood was collected *via* jugular venipuncture directly into vacutainer tubes with (plasma, whole blood) or without (serum) heparin, and stored on ice for transport back to the laboratory. Immediately upon arrival at the laboratory, blood was centrifuged at 5000 × *g* for 10 min; plasma and serum pipetted off the cellular layer into sterile microcentrifuge tubes and stored at −80°C until analysis. In addition, 1.5 ml of whole blood, collected in tubes with heparin, was aliquoted into separate, sterile microcentrifuge tubes and incubated at 37 C for 72 h. After 72 h, plasma was pipetted off the cellular layer and stored at 4 C until cytokine analysis 24–72 h later (23). Samples collected within 3 days of each other were all processed on the same cytokine assay; therefore, samples collected 3 days prior to running the assays were stored at 4 C for 72 h, samples collected 2 days prior to running the assays were stored at 4 C for 48 h and samples collected 1 day prior to running the assays were stored at 4 C for 24 h.

#### Cohort Buffalo

Cohort buffalo were originally captured in 2001 from the North of KNP and have been maintained since then in the enclosure as a BTB free breeding herd. During our study period (2014–2016), the herd included 65–70 animals. Natural births and deaths occurred during the study, leading to a total of 77 individuals included in analyses.

The enclosure is entirely within KNP and has numerous other wild animals typical of the ecosystem (e.g., giraffe, zebra, warthogs, small mammals, and small predators). However, the enclosure excludes megaherbivores (rhino, hippo, elephant) and large predators (lion, leopard). Cohort buffalo graze and breed naturally and find water in seasonal pans and manmade (permanent) water points. In extreme dry seasons, supplemental grass and alfalfa hay are supplied.

Cohort buffalo were caught every 2–3 months from February 2014 to February 2016, totaling 10 capture periods. To sample, buffalo were herded into a capture corral, separated into groups of 4–10 animals, and sedated. Buffalo that evaded corral capture were darted individually from a helicopter. Sedation procedures are outlined in Couch et al. (24). Animals were released from the capture corral within 1–5 days after captures.

The animals’ sex was determined visually. Age was determined by a combination of incisor wear and tooth emergence for animals older than 2.5 years, and *via* body size and horn growth in younger calves, as described in Jolles et al. (25). Body condition was determined by assigning a score from 1 to 5 based on manually palpating four sites (ribs, hips, spine, and tail base); average score was used in all analyses (26). At each capture period, blood was collected and processed identically to FMDV experiment procedures, with the addition of serum being stored for analysis of exposure by respiratory pathogens.

### Laboratory Methods

Foot-and-mouth disease virus qRT-PCR and respiratory pathogen ELISAs were run using serum samples. NSMI markers were quantified using plasma samples; cytokine assays were run using incubated plasma samples (outlined in field methods section) whereas haptoglobin and SAA assay were run using non-incubated plasma samples.

#### FMDV Experimental Buffalo

The number of FDMV RNA genome copies per ml of serum, expressed as log_10_, was measured using quantitative qRT-PCR methods outlined in Ref. (21). Buffalo were considered to have an active viral infection if genome copies per ml of serum were >3.2 log_10_. Thus, one individual was removed from the study as serum qRT-PCR results never exceeded >3.2 log_10_ genome copies/ml of serum.

Non-specific markers of inflammation were measured *via* sandwich ELISA per manufacturers’ instructions (Haptoglobin: Life Diagnostics 2410; Serum amyloid A: Life Diagnostics SAA-11; TNF-α: Ray-Bio ELB-TNFa; IFNγ: Bio-Rad MCA5638KZZ). All NSMI ELISAs were run within 1 month of collection.

Importantly, FMDV experimental buffalo were monitored for exposure to seven common respiratory pathogens, however, no animals seroconverted during the experiment. Pathogens tested for, and methods used to estimate, sero-conversion are identical to methods outlined below (Methods, laboratory methods, cohort buffalo).

#### Cohort Buffalo

Identical to the FMDV experiment, APP were measured *via* sandwich ELISA per manufacturers’ instructions (Haptoglobin: Life Diagnostics 2410; Serum amyloid A: Life Diagnostics SAA-11; TNF-α: Ray-Bio ELB-TNFa; IFNγ: Bio-Rad MCA5638KZZ).

Sero-conversion, a proxy for incidence, of seven common viral and bacterial respiratory pathogens (Figure 1) was measured for each capture period *via* sandwich ELISAs per manufacturers’ instructions [Adenovirus (AD-3), parainfluenza virus (Pi-3), bovine herpes virus, *Mannheimia hemolytica, Mycoplasma bovis* (MB): Bio-X IPAMM; bovine diarrhea virus (BVDV): Bio-X BVDV; bovine respiratory syncytial virus: Bio-X BRSV]. Samples were considered positive for pathogen antibodies if antibody titers exceeded threshold absorbance values calculated using the quality control procedures outlined in each Bio-X kit. Incidence was calculated as a binomial variable. Incidence was assigned a 1 if an animal seroconverted from *t*_0_ to *t*_1_ (i.e., absorbance values were below threshold concentrations at *t*_0_ but above threshold absorbance at *t*_1_) and 0 if the animal had not seroconverted.

With the exception of SAA, all NSMI and respiratory pathogen ELISAs were run within 2 weeks of capture periods. All SAA ELISAs were run in September 2016.

### Statistical Analyses

#### FMDV Experimental Buffalo

Mathematical modeling was carried out using R [R Core Team (27)]. To evaluate the response of each NSMI to FMDV infection, we calculated (i) the time to NSMI peak from initial FMDV exposure (i.e., from the first day FMDV serum genome copies/1 ml of serum >3.2 log_10_), and (ii) the period for which NSMI remained elevated after the host cleared the virus. In addition, mean peak concentration and baseline concentration were calculated for each NSMI.

The period for which NSMI remained elevated past viral clearance was calculated as follows: first, an exponential decay curve Eq. 1 was fit starting from peak NSMI concentration (Figure 1):

(1) $$y=ae^{kt}.$$

Next, decay rate (*k*) and intercept (*a*) were extracted from individual exponential decay equations, and baseline NSMI (*y*_BL_) levels were estimated from averaging day-2 and day-14 NSMI concentrations. The time when NSMI returned to baseline levels after their peak, (*t*_BL_), was calculated using Eq. 2:

(2) $$t_{\text{BL}}=\frac{\text{log}(y_{\text{BL}})-\text{log}(a)}{k}.$$

Time at viral clearance (*t*_VC_) was assigned based on the first-day FMDV genome copies dropped below 3.2 log_10_/ml of serum after initial incidence. Days’ NSMI was elevated past viral clearance which was calculated by Eq. 3:

(3) $$t_{\text{NSMI elevated from vc}}=t_{\text{BL}}-t_{\text{VC}}.$$

Animals in which the NSMI concentration did not exceed twofold baseline levels were determined not to have mounted that particular NSMI response and removed from future analysis (for that NSMI). If NSMI concentrations did not peak until 30 days post FMDV challenge these animals were removed from the analysis as their exponential decay curve would have been fit to only one data point. Final sample sizes included in each NSMI analysis are included in Table 1.

**Table 1:** Foot-and-mouth disease virus (FMDV) experiment: mean (±SE) baseline non-specific marker of inflammation (NSMI) concentration, peak NSMI concentration, days from FDMV incidence to peak concentration and days elevated from viral clearance for the FMDV virus.

NSMI	#Buffalo that responded	Baseline concentration (ng/ml)		Peak concentration (ng/C)	
		Mean	SE	Mean	SE
Haptoglobin	11	401.26	22.38	491,891	22,722.45
Serum amyloid A (SAA)	11	273.46	20.02	13,806.5	135.45
TNF-α	3	0.88	0.45	3.18	1.61
IFNγ	11	0.52	0.08	7.3	0.49

**NSMI**		**Days to peak**		**Days elevated post viral clearance**	
		**Mean**	**SE**	**Mean**	**SE**

Haptoglobin		5.4	0.29	21.23	0.39
SAA		3.33	1.17	11.18	2.66
TNF-α		6.67	0.19	7.75	2.56
IFNγ		4.4	0.3	16.51	1.74

*Animals were considered to have mounted a NSMI response if NSMI concentration exceeded 2× baseline concentration after FMDV infection; with a total of 11 animals participating in the study. Days to peak was calculated by counting the number of days between the first day FMDV RNA copies exceeded 3.2 copies/5 μl of serum and the day NSMI reached peak concentration. Days elevated from viral clearance was calculated by estimating the time it took for NSMI to return to baseline concentrations after viral clearance (when FMDV RNA copies were less than 3.2 copies/5 μl serum post FDMV incidence). If peak concentrations were only reached on day 30, animals were excluded from mean calculations of days to peak and days elevated from viral clearance. Notably, mean peak concentration for haptoglobin was approximately 1,226 times higher than mean baseline levels, 50 times higher for SAA, 14 times higher for IFNγ, and four times higher for TNF-α*.

Raw data of NSMI concentration by day is presented in SF1.

#### Cohort Buffalo

Statistical analyses for cohort buffalo were performed in R using lme4 (28) and lmerTest (29).

Mixed effects logistic regressions were used to evaluate the effect of NSMI on respiratory disease incidence. Multiple samples per individual were used for all analyses, thus Animal ID was included as a random intercept to avoid pseudo-replication. Host traits (body condition, age, sex) and season may influence respiratory disease incidence (30); therefore, they were included as fixed effects within each model. A model was run for each combination of respiratory pathogen × NSMI (mixed effects logistic regression model example Eq. 4):

(4) $$\begin{array}{l} \text{logit}\left\{P\left({\text{incidence}}_{ij}=1{\text{|NSMI}}_{1j},{\text{Body Condition}}_{2j},{\text{sex}}_{3j},{\text{age}}_{4j},\varsigma_{j}\right)\right\}\\ \;=\beta_{0}+\beta_{1}*{\text{NSMI}}_{1j}+\beta_{2}*\text{Body Condtion}+\beta_{3}*\text{sex}+\beta_{4}*\text{age}+\beta_{5}*\text{season}+\varsigma_{j} \end{array}$$

where ς_*j*_ ~ *N*(0,ψ) represents Animal ID as a random intercept.

The association of NSMI with respiratory disease incidence was evaluated post sero-conversion. Our models asked whether prior disease incidence between [*t*_0_ and *t*_1_] was associated with elevated NSMI at *t*_1_. Thus, each model was run with explanatory variables corresponding to the *t*_1_ time step, and disease incidence measured for the preceding capture interval.

Haptoglobin and SAA spanned several orders of magnitude and were severely right skewed, thus were log_2_ transformed to increase model stability and avoid issues with influential data points.

To prevent errors that can arise from multiple testing, statistical significance of each dependent variable was defined using significance levels corrected via the Benjamini and Hochberg’s false discovery rate controlling procedure (31). Benjamini and Hochberg’s false discovery rate controlling procedure assigns a significance level based upon rank of *p*-value within the family of tests; therefore, the particular significance level for each model is specified within Table 2. The test statistic and resulting *p*-value were calculated using Satterthwaite’s approximation of degrees of freedom (29).

**Table 2:** Cohort study: results of logistic regression models examining the non-specific markers of inflammation (NSMI) as indicators of recent (2–3 months) parasite exposure after accounting for body condition, sex, age, season, and animal id.

NSMI	Pathogen	β	SE	Test statistic	FDR sig level	*p*-Value
**log_2_ (Haptoglobin)**						
	Bovine herpes virus (BHV)	−0.06307	0.038	−1.66	0.021	0.09695
	PI-3	0.12049	0.03749	3.214	0.007	**0.00131**
	Bovine respiratory syncytial virus (BRSV)	−0.05834	0.05671	−1.029	0.043	0.655834
	Bovine diarrhea virus (BVDV)	−0.03116	0.03953	−0.788	0.029	0.4309
	AD-3	0.01374	0.03651	0.376	0.05	0.707
	Mycoplasma bovis (MB)	0.19008	0.0598	3.178	0.014	**0.00148**
	*Mannheimia hemolytica* (MH)	−0.02125	0.03593	−0.592	0.036	0.55414

**log_2_ (Serum amyloid A)**						
	BHV	−0.04368	0.02235	−1.954	0.007	0.0507
	PI-3	0.003432	0.019632	0.175	0.05	0.86123
	BRSV	−0.03458	0.03394	−1.019	0.036	0.308322
	BVDV	−1.32E–01	8.95E−02	−1.471	0.029	0.14131
	AD-3	0.01316	0.02144	0.614	0.043	0.53946
	MB	0.3577	0.19031	1.88	0.014	0.060174
	MH	−0.04644	0.02661	−1.745	0.021	0.0809

**TNF-α**						
	BHV	−0.19076	0.3101	−0.615	0.036	0.5384
	PI-3	−0.32148	0.26095	−1.232	0.007	0.21798
	BRSV	−0.62706	71187	−0.881	0.021	0.378
	BVDV	−0.24408	0.35926	−0.679	0.029	0.496886
	AD-3	−0.099964	0.212616	−0.47	0.043	0.638
	MB	0.277238	0.22845	1.192	0.014	0.233131
	MH	−0.064314	0.235515	−0.273	0.05	0.784793

**IFNγ**						
	BHV	−0.77378	0.58834	−1.315	0.029	0.18844
	PI-3	0.20225	0.18011	1.123	0.043	0.26148
	BRSV	−3.63397	2.19124	−1.658	0.021	0.0972
	BVDV	0.17317	0.1457	1.189	0.036	0.234609
	AD-3	0.34937	0.18254	1.914	0.014	0.0556
	MB	−2.23E+00	1.03E + 00	−2.157	0.007	0.031
	MH	0.26631	0.29246	0.125	0.05	0.900229

*FDR significance levels are false discovery rate significance levels, which avoid issues for false positives that can occur when using multiple testing procedures*.

*Bold values indicate statistically significant relationships*.

For significant associations between pathogen incidence and NSMI, average marginal predicted probabilities for given levels of NSMI concentration and area under the curve (AUC) were calculated using R packages lme4 (28) and pROC (32). Marginal predicted probabilities were calculated using models described in Eq. 4. 1,000 marginal predicted probabilities of pathogen incidence were calculated for 100 fixed values of NSMI and randomly selected (from the data) values of age, sex, body condition, season, and animal id. Average marginal predicted probability and 95% CI intervals for pathogen incidence were then constructed from the 1,000 values calculated for each fixed NSMI concentration. AUC, or the area under the receiving operating characteristic curve, is a standard diagnostic analysis used to measure how well a parameter can distinguish between two diagnostic groups based upon the specificity (true negative rate) and sensitivity (true positive rate) of the test.

## Results

### FMDV Experiment Buffalo

**Figure 2:**
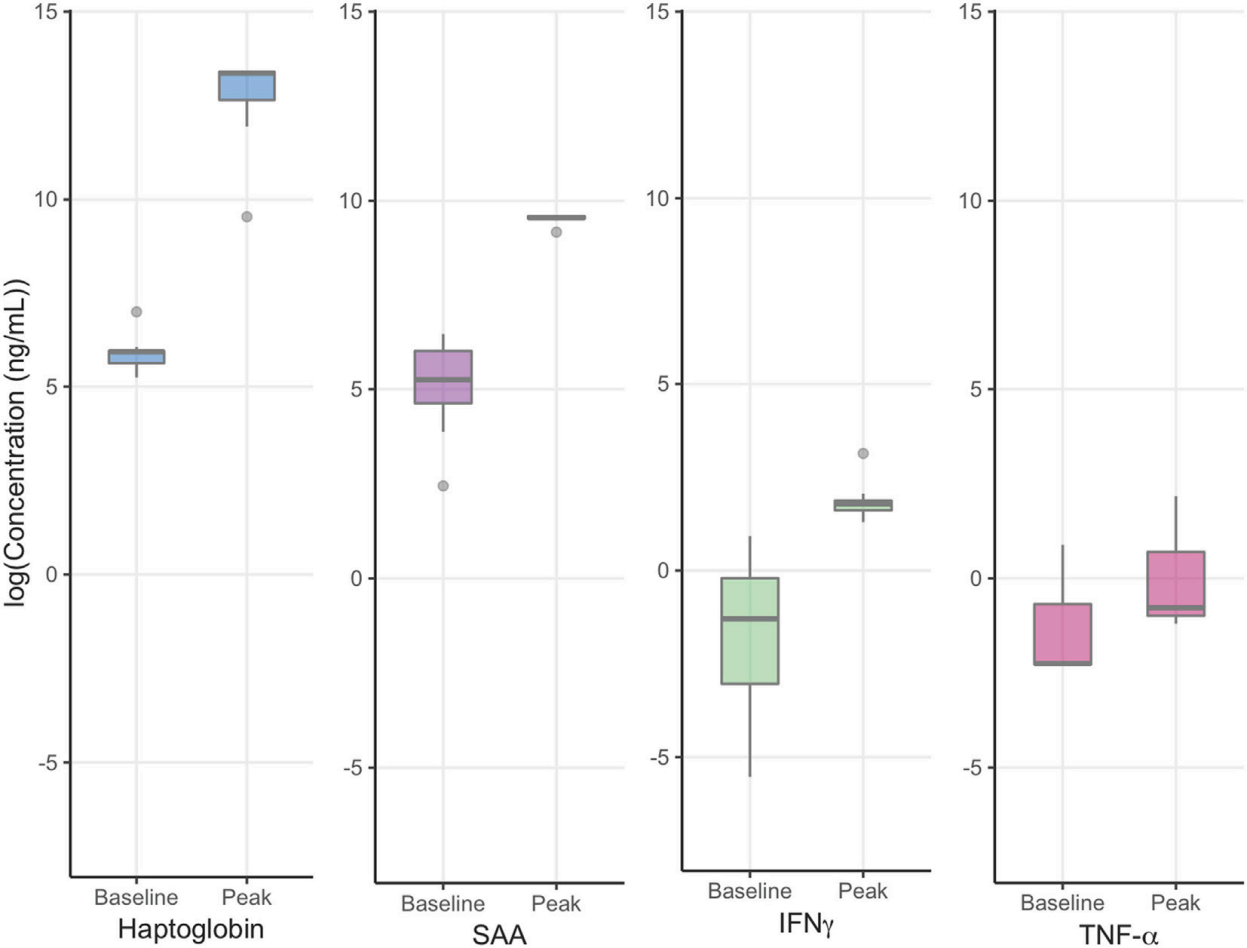
Foot-and-mouth disease virus experiment: mean baseline and peak NSML. *Y* axes are log transformed for ease of visual comparison between non-specific markers of inflammation peak and baseline concentrations. Haptoglobin peak and baseline concentrations displayed the greatest difference and least variability followed by serum amyloid A (SAA), IFNγ, and TNF-α. The horizontal bands represent the 25, 50, and 75% quartiles whereas the vertical lines represent 1.5 times the interquartile range above the upper quartile and below the lower quartile, and dots represent outliers.

Buffalo mounted robust NSMI responses to FMDV infection, as evidenced by differences between mean peak and baseline NSMI concentrations (Table 1; Figure 2).

**Figure 3:**
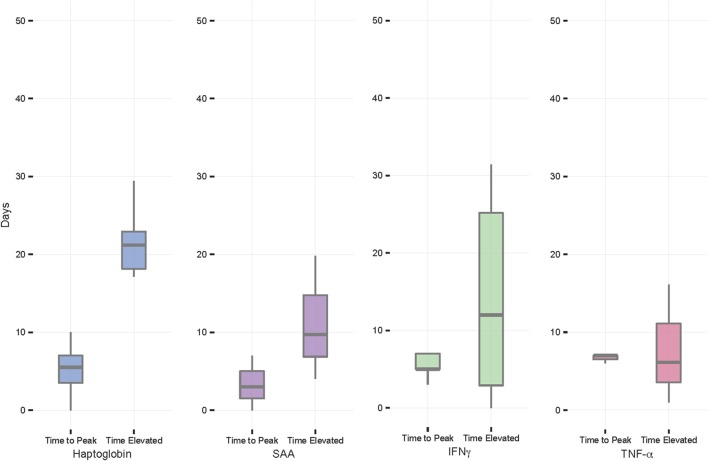
Foot-and-mouth disease virus experiment: time from initial FMDV incidence to peak non-specific markers of inflammation concentration and time NSMI elevated from viral clearance. On average, all NSMI concentrations reached peak in 3–7 days. Haptoglobin concentrations remained elevated the longest past viral clearance, with the least variability, followed by serum amyloid A (SAA), IFNγ, and TNF-α. Individuals where NSMI concentrations peaked on day 30 were excluded from calculations as this was thought to be due to a secondary infection. The horizontal bands represent the 25, 50, and 75% quartiles, whereas the vertical lines represent 1.5 times the interquartile range above the upper quartile and below the lower quartile, and dots represent outliers.

The mean time from FMDV incidence to peak NSMI concentration was 3–7 days for all NSMI (Table 1; Figure 3). On average, viral clearance occurred at 4.72 (±0.20) days after initial FMDV infection (i.e., the first-day FMDV RNA copies >3.2 log_10_/ml of serum). Haptoglobin remained elevated for the greatest number of days past viral clearance (21 days on average), with the lowest interindividual variation in time elevated, followed by IFNγ, SAA, and TNF-α (Figures 1 and 3; Table 1).

All individuals showed increases in haptoglobin, SAA, and IFNγ, however, only 3/11 contact buffalo mounted detectable TNF-α responses. Haptoglobin displayed the greatest difference in mean peak and baseline concentration, followed by SAA, IFNγ, and TNF-α.

### Cohort Buffalo

**Figure 4:**
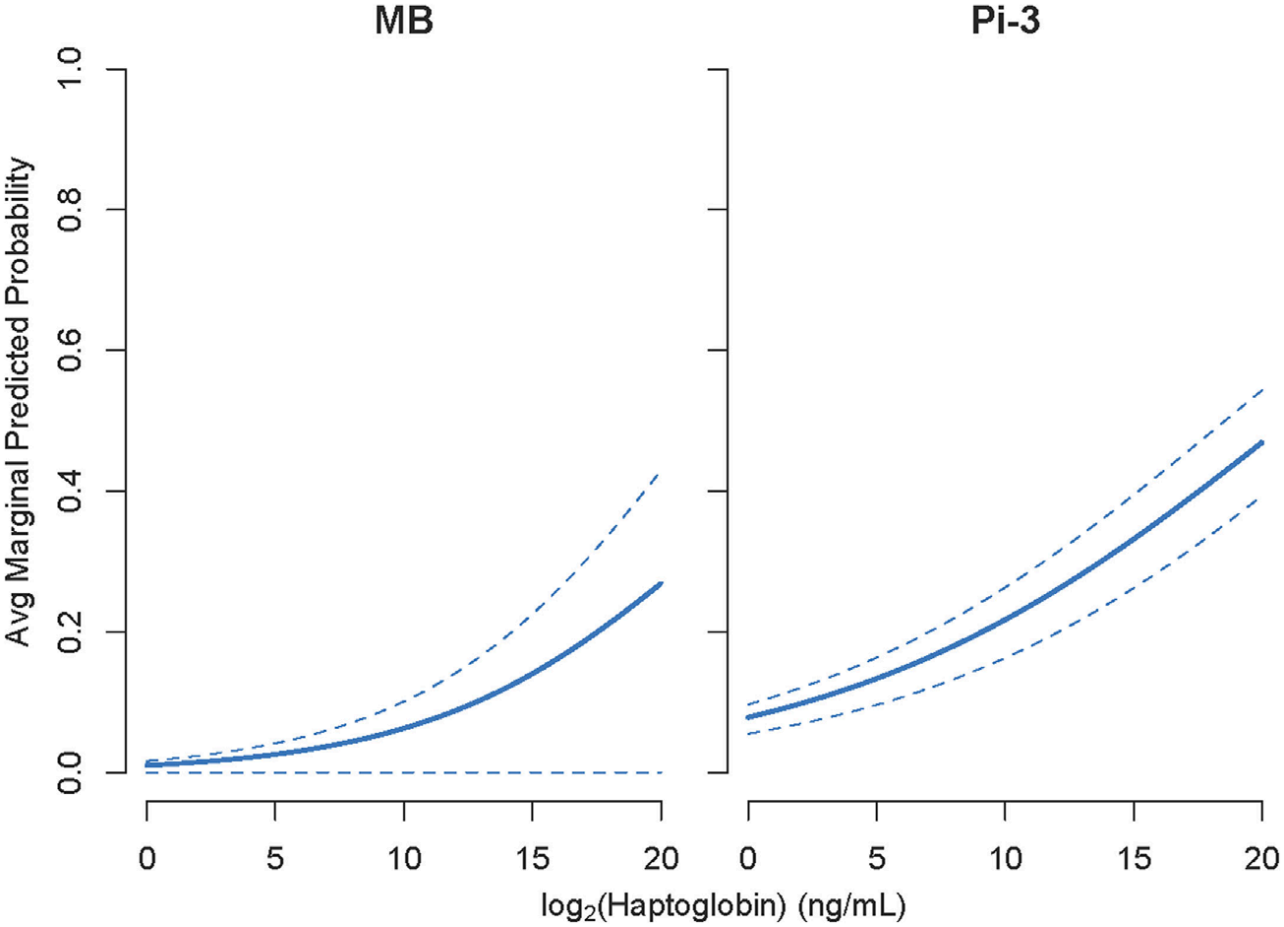
Cohort study: elevated haptoglobin was associated with *Mycoplasma bovis* (MB) and *Parainfluenza virus III* (PI3) exposure during the preceding 2–3 months. *Y* axes show average marginal predicted probabilities of pathogen incidence. Marginal predicted probabilities were calculated using models described in Eq. 4. 1,000 marginal predicted probabilities of pathogen incidence were calculated for 100 fixed values of NSMI and randomly selected (from the data) values of age, sex, body condition, season, and animal id. Average marginal predicted probability and 95% CI intervals for parasite incidence were then constructed from the 1,000 values calculated for each fixed NSMI concentration. Due to large seasonal variation, the lower confidence interval of MB is small.

**Figure 5:**
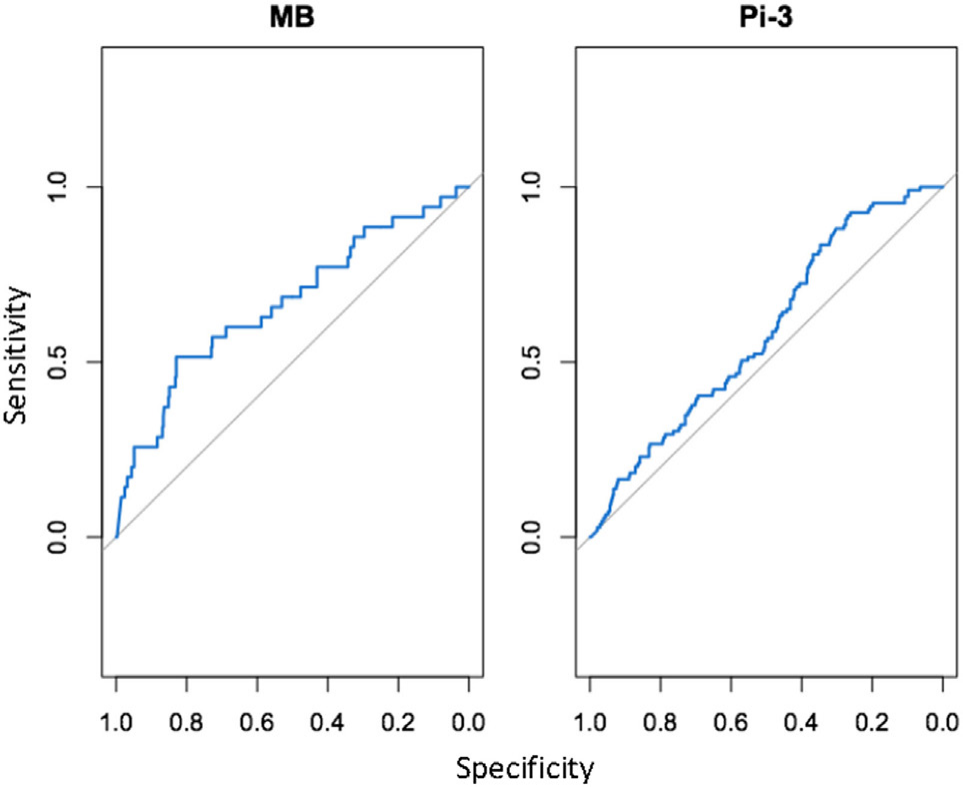
Cohort study: area under the curve (AUC) for detection *of Mycoplasma bovis* (Mb) and *Parainfluenza Virus* (PI3) based on elevated haptoglobin. AUC, or the area under the receiving operating characteristic (ROC) curve, is a standard diagnostic analysis used to measure how well a parameter can distinguish between two diagnostic groups based on the specificity (true negative rate) and sensitivity (true positive rate) of the test. The gray line represents the trend the diagnostic parameter would follow if the AUC was equal to 0.5. The blue line represents the observed trend; the closer the curve follows the left and top border of the graph, the more accurate the test. If the blue line falls below the gray line (AUC < 0.5), it indicates that the test is not significantly better than random.

For each NSMI, we tested whether elevated levels of the marker were indicative of infection by a range of respiratory pathogens during the preceding 2–3 months. Haptoglobin was a significant indicator of two respiratory pathogens: MB and Pi-3 (Table 2; Figure 4). After controlling for animal traits and season, for every twofold increase in haptoglobin there was a 21% increase in the odds of prior MB incidence and a 13% increase in the odds of prior Pi-3 incidence. As expected for NSMI, the sensitivity and specificity of haptoglobin as a marker of each particular pathogen was significant (Lower CI of AUC > 0.5) but moderate. The AUC for haptoglobin as a classifier of MB was 0.67 (95% CI 0.52–0.77) and Pi-3 was 0.586 (95% CI 0.53–0.64) (Figure 5).

Although not significant by standards of the Benjamini and Hochberg’s false discovery rate controlling procedure, there was suggestive evidence (*p*-value <0.05) that IFNγ was an indicator of MB incidence (Table 2). For every unit increase in IFNγ, there was an 11% decrease in the odds of prior MB incidence.

## Discussion

Mitigating disease outbreaks and identifying pathogen presence is crucial in evaluating ecosystem health (33, 34), creating effective wildlife conservation plans (35–37) and improving global health (38–40). Current techniques to detect pathogen exposure are primarily limited to (1) tests that are highly specific to both pathogen and host and (2) pathogens that cause detectable pathology in humans and economically important animals; yet, the diversity of pathogen communities in natural populations is only beginning to be uncovered (41, 42) with specific diagnostic tools for novel infections generally unavailable.

Given the current surge in infectious disease emergence (43), new diagnostic approaches, which can detect diverse pathogens, over an extended time frame within a broad range of hosts, are urgently needed. Our study demonstrates a possible approach to detecting infections non-specifically, using inflammatory molecular.

Despite the overwhelming diversity of pathogen species that can infect a given host, early stages of immunological response are considered evolutionarily conserved, and primary defenses are similar for a diversity of pathogens (44) within many hosts (45). Consequently, tracking first-line immune response has potential as a non-specific diagnostic approach for monitoring the burden of disease in a population of interest. Invertebrate and vertebrate hosts initially respond to pathogen challenge by mounting an inflammatory response (45). Due to the ubiquity of the inflammatory response, proteins upregulated during this initial stage of infection may hold promise as non-specific markers of pathogen exposure.

In this study, we used experimental and observational approaches to explore the utility of four NSMI in detecting pathogen exposure. We included two APP (haptoglobin and SAA) and two cytokines involved in inflammatory responses (IFNγ and TNF-α).

Buffalo mounted quick and robust acute phase responses to experimental challenge with FMDV, with the magnitude of NSMI responses similar to those reported in cattle (46). We found that, in response to FMDV infection, haptoglobin remained elevated the greatest number of days past viral clearance with the smallest degree of interindividual variation. Haptoglobin reached peak concentrations within a week of FMDV incidence and remained elevated for more than 3 weeks past FMDV clearance. Elevated haptoglobin levels were, thus, detectable both during and for several weeks after FMDV infection. Complementary to this, we found in our cohort study that haptoglobin was a significant indicator of recent natural incidence by two out of seven viral and bacterial respiratory pathogens.

Within the last 20 years, haptoglobin has been used to study inflammation in domestic animals (46) but has been more strongly associated with bacterial infections (47). We found haptoglobin to be significantly associated with both a viral (Pi-3) and a bacterial (MB) pathogen. Abnormal haptoglobin concentrations have been found in cattle infected with FMDV (48, 49) and Pi-3 (50).

All buffalo included in the experimental study mounted SAA and IFNγ responses to experimental FMDV infection within a week, however, on average, SAA remained elevated for just under 2 weeks and IFNγ remained elevated for just over 1 week. IFNγ was also a suggestive indicator of MB in our cohort study. TNF-α responses were detectable in one-fourth of our experimentally FMDV-infected buffalo, were short-lived for animals that mounted a response, and showed no associations with respiratory pathogens we monitored in our cohort study. Our results for SAA and IFNγ, especially IFNγ, suggest potential of NSMI for disease monitoring. Perhaps, inflammatory cytokines, particularly TNF-α, responses are mounted quickly, either very localized or low in magnitude, and short lived because of the collateral damage they elicit (51, 52). Haptoglobin contributes to “cleaning up” products of inflammation (19) and, thus, should cause significantly less immunopathology. The function of haptoglobin may, thus, explain the comparatively long lived, high magnitude responses we observed.

We found haptoglobin to be a significant classifier of MB and Pi-3, however, specificity would be considered low by veterinary and human medical diagnostic standards. Low specificity is expected, given that haptoglobin responds to multiple inflammatory processes including exposure to unknown pathogens, stress, trauma, and autoimmune disorders (46); and indeed, the goal here was to find non-specific markers indicative of pathogen exposure. Although sensitivity and specificity was low, and haptoglobin only detected two out of seven respiratory pathogens, our results are particularly encouraging because we are likely to be underestimating the true sensitivity of haptoglobin and other NSMI in the cohort study, due to the “mismatch” between capture interval (2–3 months) and NSMI response (e.g., haptoglobin: 3 weeks). This is likely cause an increased number of false negatives—animals that were exposed to a given pathogen, but have no detectable elevation in NSMI at time of capture. More frequent captures should thus improve the performance of NSMI in detecting pathogen exposures. In addition, using a combination of NSMI may help to tease apart sources of inflammation, allowing researchers to filter out non-infectious processes and improve test specificity.

Our work points to the possibility of defining markers for non-specific disease surveillance, but raises many new questions about discovering which combinations of markers can potentially work in different host species, and for detection of different suites of pathogens.

For example, future research could investigate a broader range of cytokines, such as inflammatory cytokines, Il-6 and Il-1beta, and additional APP, such as fibrinogen or C-reactive protein, and negative APP such as albumin or transferrin. Dugovich et al. (53) recently described the utility of natural antibodies (nAbs), antibodies that associate with the innate immune response and bind to multiple microbial agents, in assessing immunological status of desert bighorn sheep. In addition, in mammals, toll-like receptors (TLRs), proteins integral in recognition of infection, are highly conserved to recognize broad groups of pathogens (54). As such, the utility of nABs and TLR expression as disease surveillance tools warrants future research.

A systematic approach could follow host responses to pathogenic challenge, from pathogen recognition to inflammation, and define effectors that typify responses to different groups of pathogens. Immunologists could potentially tailor NSMI panels for detecting different groups of parasites, such as hemoparasites or gastro-intestinal infections—and explore whether taxonomic relatedness of parasites, or similarity of infection sites are most important in selecting appropriate NSMI.

Assays for APP and pro-inflammatory cytokines have been developed for domestic animals and laboratory model species, including cows, sheep, goats, horses, dogs, cats, mice, and rats. A handful of studies have used serum and urine based assays to monitor health and disease incidence in wildlife species including Grant’s zebra (55), European mouflon (56), Przewalkski’s horses (57), rhesus macaques (58). As such, the tools for beginning to define panels of NSMI for disease monitoring, already exist for a broad range of mammalian host species. Due to the devastation that emerging infectious diseases have elicited in amphibian (59) and marine invertebrate (60, 61) systems, identifying inflammatory markers that detect pathogen exposure in non-mammalian vertebrates and invertebrates could prove invaluable to conservation biologists.

For NSMI that are stable in stored samples, such as frozen sera, the utility of NSMI could extend beyond current surveillance to include retrospective studies—biobanks are a commonly available but underused resource for human, animal, and wildlife studies. Beechler et al. (62) demonstrated that haptoglobin concentrations in stored serum remain stable for at least 4 years, and (63) documented stability of haptoglobin, nAbs, and total immunoglobulins during extended storage, suggesting that undertaking retrospective evaluations of populations is a feasible and viable option for future studies.

Developing non-specific diagnostic tools is essential to detect emerging infections in animal and human populations and effectively tracking the burden of infection in natural populations. In the face of the vast diversity of pathogens and host species, an approach that tracks conserved inflammatory responses to a range of infections may provide a tractable pathway toward recognizing changes in disease burden that can then be followed up with specific diagnostic testing. Our study on infections in African buffalo provides a proof of concept, showing that APP and/or pro-inflammatory cytokines can provide useful information about pathogen exposures. It is our hope that this work will open opportunities for investigating suites of NSMI as indicators for pathogen exposure, potentially transforming how we measure disease in natural populations.

## Ethics Statement

The study was conducted under South Africa Department of Agriculture, Forestry and Fisheries Section 20 permits Ref 12/11/1 and Ref 12/11/1/8/3, Oregon State University ACUP project number 4478 and 4861, Onderstepoort Veterinary Research Animal Ethics Committee project number 100261-Y5, and the Kruger National Park Animal Care and Use Committee project number JOLAE1157-12 and JOLAE1157-13.

## Author Contributions

AJ: project PI, PhD advisor of lead author CG, involved in idea, field work, lab work, analyses, and manuscript writing. CG: lead author involved in field work, lab work, leads analyses, and manuscript writing. BB: involved in idea, field work, lab work, analyses, and manuscript writing. PB: scientific liaison for South African National Parks Veterinary Wildlife Services. Involved in project design and field work. BC: Project PI for the Pirbright Institute. Involved in project design, lab work, and field work. LK-L: state veterinarian involved in project design, lead experimental study, and manuscript preparation. FM: involved in diagnostic lab work, experimental study design and implementation, and manuscript preparation. TM: involved in data analysis. EP-M: involved in field work, lab work, and manuscript preparation. KS: involved in field work, lab work, and manuscript preparation. OS: state veterinarian involved in project design, lead experimental study, and manuscript preparation.

## Conflict of Interest Statement

The authors declare that the research was conducted in the absence of any commercial or financial relationships that could be construed as a potential conflict of interest.
