# Supplementary figures and images for: Detection of Pathogen Exposure in African Buffalo Using Non-Specific Markers of Inflammation
Source: Front Immunol. 2018 Jan 11;8:1944. doi: 10.3389/fimmu.2017.01944 (PMC5768611; doi:10.3389/fimmu.2017.01944)

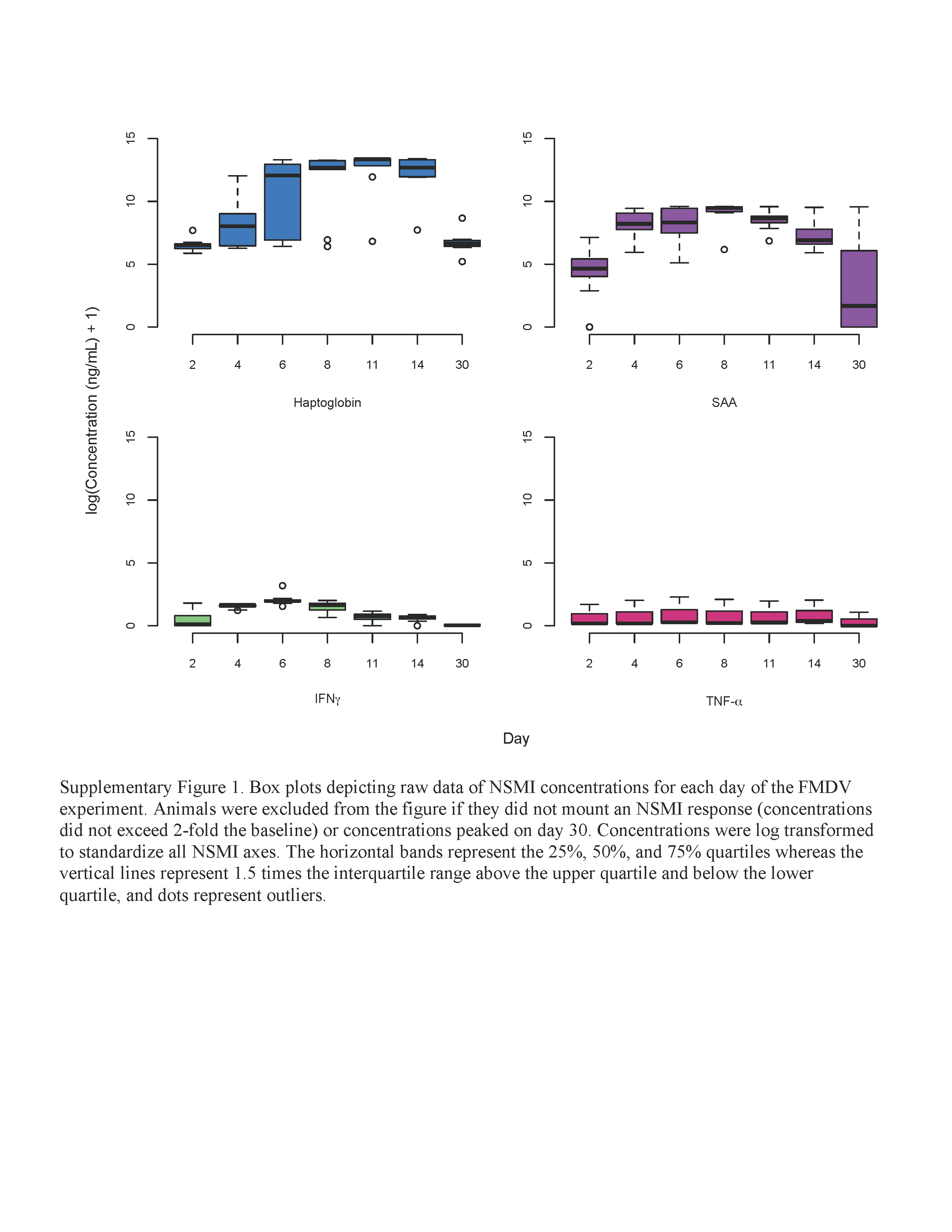

Supplement: Supplementary file 1 [file Image_1.tiff]
